# Immunomodulatory effects of QsCATH on macrophages: transcriptomic insights and molecular docking analysis

**DOI:** 10.1038/s41598-025-28482-9

**Published:** 2025-12-12

**Authors:** Fen Qiao, Xin-Yi Qian, Jia-Le Wu, Zi-Xuan Wang, Yi-Kai Feng, Jie Chen

**Affiliations:** 1 College of Ecology, Lishui University, Lishui, 323000 China; 2 Industrial College of Traditional Chinese Medicine and Health, Lishui University, Lishui, 323000 China

**Keywords:** Amphibian antimicrobial peptide, QsCATH, Macrophages, Transcriptomics, Molecular docking, Immunomodulation

## Abstract

The molecular mechanisms underlying the immunomodulatory effects of cathelicidins on macrophages remain poorly characterized. This study aimed to elucidate the immunomodulatory mechanisms of QsCATH, an antimicrobial peptide derived from the Chinese spiny frog (*Quasipaa spinosa*), in RAW264.7 macrophages using RNA sequencing and molecular docking. Transcriptomic analysis revealed that QsCATH significantly downregulated inflammatory-related genes (*tnf*, *il6*, *ccl5*, *il1b*) and suppressed pro-inflammatory cytokine production (tumor necrosis factor (TNF)-α, interleukin (IL)-6, IL-1β) by inhibiting key pathways such as NF-κB, TNF, and NOD-like receptor signaling. Kyoto Encyclopedia of Genes and Genomes and Gene Ontology enrichment analyses showed that QsCATH balanced inflammatory resolution with host defense by modulating biological processes including “immune system processes,” “defense responses,” and “oxidative stress responses.” Using molecular docking simulations, potential interactions were predicted between QsCATH and membrane-associated proteins Nod2, Ripk2, and Itga3 (binding scores: − 239.00, − 213.24, and − 290.08, respectively), suggesting direct interference with receptor-mediated signaling cascades. This study presents the first transcriptome-level analysis of the immunoregulatory network of amphibian cathelicidins, providing insights for developing novel peptide-based therapeutics against drug-resistant infections and inflammatory disorders.

**Supplementary Information:**

The online version contains supplementary material available at 10.1038/s41598-025-28482-9.

## Introduction

Antimicrobial peptides (AMPs) are evolutionarily conserved components of innate immunity, serving as dual-function molecules capable of directly neutralizing pathogens and orchestrating host immune responses^1^. Among these, cathelicidins represent a phylogenetically ancient family with roles in bridging innate and adaptive immunity through chemotaxis modulation, cytokine regulation, and macrophage polarization^2–5^. Vertebrate cathelicidins, such as human LL-37, exhibit broad immunomodulatory effects, including pro-inflammatory cytokines (tumor necrosis factor (TNF)-α, interleukin (IL)-6) suppression during sepsis, and bacterial clearance enhancement via phagosome maturation^6–8^. This paradigm has been expanded by recent discoveries of amphibian-derived cathelicidins, particularly those from Chinese spiny frogs (*Quasipaa spinosa*), revealing structural innovations such as amphipathic α-helical domains that enhance membrane permeability^5,9,10^. However, despite their therapeutic potential, the molecular mechanisms underlying the immunoregulatory effects of these cathelicidins on macrophages (the sentinel immune cells) remain largely unexplored, particularly at the systems biology level.

Macrophages, which are central coordinators of innate immunity, employ a sophisticated group of pattern recognition receptors, including Toll-like receptors (TLRs), NOD-like receptors (NLRs), and integrins, to detect pathogen-associated molecular patterns^11,12^. Activation of these receptors triggers canonical signaling cascades, such as NF-κB and MAPK pathways, which regulate cytokine production, such as TNF, IL-1β, and chemokines (CCL5, CXCL2) critical for resolving inflammation^13^. Emerging evidence suggests that certain AMPs modulate these pathways through direct receptor interactions. For example, porcine β-defensin-2 interacts with TLR4 and attenuates lipopolysaccharide (LPS)-induced NF-κB activation^14^, while cathelicidin and LPS act synergistically to promote LPS endocytosis and endosomal TLR4 activation, inducing chemokine CXCL8/CXCL1 secretion by intestinal epithelial cells, which enhances neutrophil recruitment^15^. Paradoxically, excessive AMP-mediated immunomodulation may disrupt immune homeostasis, as shown by the dual role of LL-37 in exacerbating autoimmune diseases while protecting against microbial invasion^16^. These findings highlight the need for precise characterization of AMP-macrophage interactions, particularly for non-mammalian peptides like QsCATH, whose structural divergence may confer unique target specificity^5,9^.

The focus of current research on amphibian AMPs is predominantly on their direct antimicrobial properties, with limited exploration of their immunoregulatory networks^17^. Traditional approaches, such as cytokine profiling and receptor knockout models, provide fragmented insights; however, system-wide molecular dynamics are not captured. To address this gap, next-generation sequencing technologies coupled with bioinformatics pipelines now enable comprehensive transcriptomic analyses, revealing AMP-induced perturbations in immune gene networks^18^. For example, transcriptome studies of Epinecidin-1 reveal that it inhibits pro-inflammatory signals, increases anti-inflammatory responses, and enhances antigen presentation capabilities, creating a multi-layered network of immune regulation. This method alleviates LPS-induced inflammatory damage and could potentially improve the host’s defense mechanisms against pathogens^19^.

In this study, RNA-seq was integrated with molecular docking to investigate how QsCATH modulates immunity induced by RAW264.7 macrophages. Transcriptomic profiling was used to identify key regulatory pathways (NF-κB, TNF, NOD signaling), while structural modeling was used to predict QsCATH interactions with potential receptors. We provide a systems-level understanding of amphibian cathelicidins by bridging omics and structural data, advancing peptide therapeutic design against drug-resistant infections and inflammatory disorders (Table 1).

**Table 1:** Primer sequences used in this study.

Accession number	Gene	Sequence
NM_013590	*lyz1*	F: GCTTCTACTGCAGCCCATTC
		R: GCTGACTGACAAGGGAGACT
AY160221	*nod2*	F: GTGTTTGGGGCTGTCAGAAG
		R: GAGACGACGTGAAGATTGGC
AF487539	*ripk2*	F: ACAGCTGGGATGGTATCGTT
		R: AAGGCAGGCTTCAGTCATCT
BC062205	*itga3*	F: TTCCACGGCTTCTTCTCCAT
		R: GGAAGTGGACCTCAGTGTGA
BC137720	*tnf*	F: GACCCCTTTACTCTGACCCC
		R: AGGCTCCAGTGAATTCGGAA
BC138766	*il6*	F: ACAAAGCCAGAGTCCTTCAGA
		R: TGGTCCTTAGCCACTCCTTC
BC033508	*ccl5*	F: TGCCAACCCAGAGAAGAAGT
		R: AGATGCCCATTTTCCCAGGA
NM_008361	*il1b*	F: AGAAGCTGTGGCAGCTA
		R: TGAGGTGCTGATGTACCA
BC119511	*cxcl2*	F: ACTCTCAAGGGCGGTCAAAA
		R: CATCAGGTACGATCCAGGCT
AY090567	*nos2*	F: CCCCGCTACTACTCCATCAG
		R: CCACTGACACTTCGCACAAA
X00686	*18S rRNA*	F: TTTGTTGGTTTTCGGAACTGA
		R: CGTTTATGGTCGGAACTACGA

## Results

### Transcriptome sequencing and assembly

To elucidate the receptor proteins and associated regulatory pathways of QsCATH on macrophages, RAW264.7 cells were subjected to QsCATH treatment for 12 h, followed by transcriptome sequencing. Sequencing using the MGISEQ-2000RS platform resulted in generating 39.93 Gb of data (Table 2). Valid data were subsequently submitted to the NCBI Sequence Read Archive (SRA) database (accession number PRJNA1220286).

**Table 2:** Quality control data for samples.

Group	Sample	Raw data/Gb	Valid data/Gb	*Q20/*%	*Q30*/%	GC ratio/%
Treatment (n = 3)	QsCATH1	6.37	6.10	99.36	97.85	51.57
	QsCATH2	7.25	6.94	99.39	98.00	51.66
	QsCATH3	6.47	6.13	99.38	97.93	51.76
Control (n = 3)	Control1	7.44	7.04	99.41	98.04	51.63
	Control2	6.36	6.07	99.40	98.01	51.64
	Control3	6.05	5.80	99.40	97.99	51.69

In total, 285,849,948 raw reads were generated from the transcriptome sequencing of three treatment and control groups. After filtering the data, 45,543,538, 51,735,266, 46,165,390, 53,081,344, 45,293,176, and 43,133,026 reads (each with a mapping rate > 95%) were respectively obtained from the three treatment and control groups (Table 3).

**Table 3:** Details of RNA sequence reads in QsCATH-treated RAW264.7 cells.

Group	Sample	Clean reads	Filtered reads	Mapping rate (%)
Treatment (n = 3)	Treatment1	45,630,608	45,543,538	95.86
	Treatment2	51,880,694	51,735,266	95.92
	Treatment3	46,290,390	46,165,390	95.74
Control (n = 3)	Control1	53,220,852	53,081,344	95.39
	Control2	45,498,502	45,293,176	95.55
	Control3	43,315,624	43,133,026	95.48

### Screening for differentially expressed genes

**Fig. 1:**
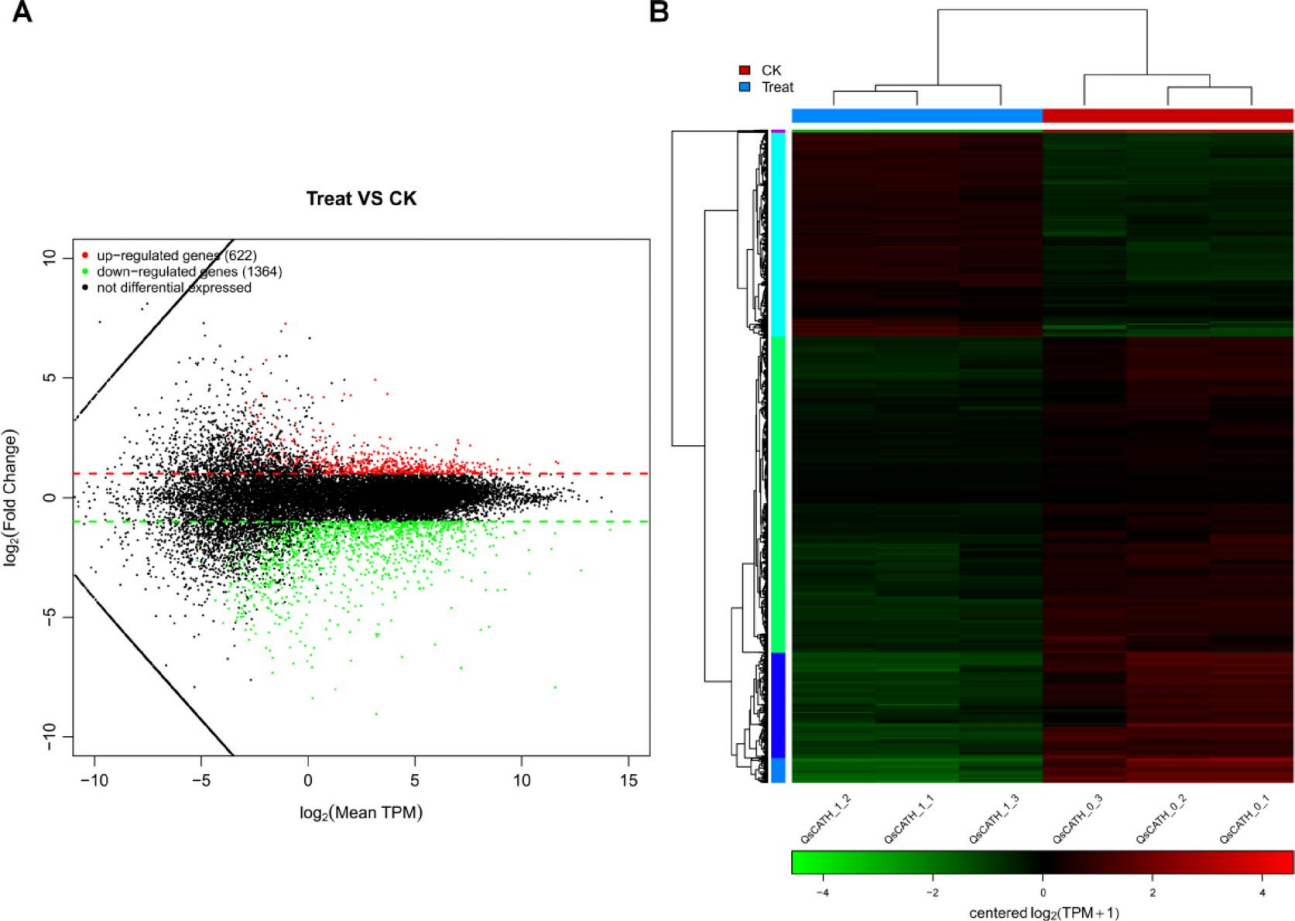
Differentially expressed genes (DEGs) in the QsCATH-treatment and untreated (control) groups of RAW264.7 cells. (**A**) Volcano plot of DEGs. (**B**) Clustering thermogram of DEGs.

DESeq analysis was used to identify 1986 genes that showed differential expression in the QsCATH-treatment and control groups. In the QsCATH-treatment group, there were 622 genes that showed increased expression while 1364 genes showed decreased expression compared with those in the control group (Fig. 1A). The three biological samples from each of the control and treatment groups were clustered together (Fig. 1B).

### KEGG analysis reveals immune-responsive pathways are altered by QsCATH in macrophages

**Fig. 2:**
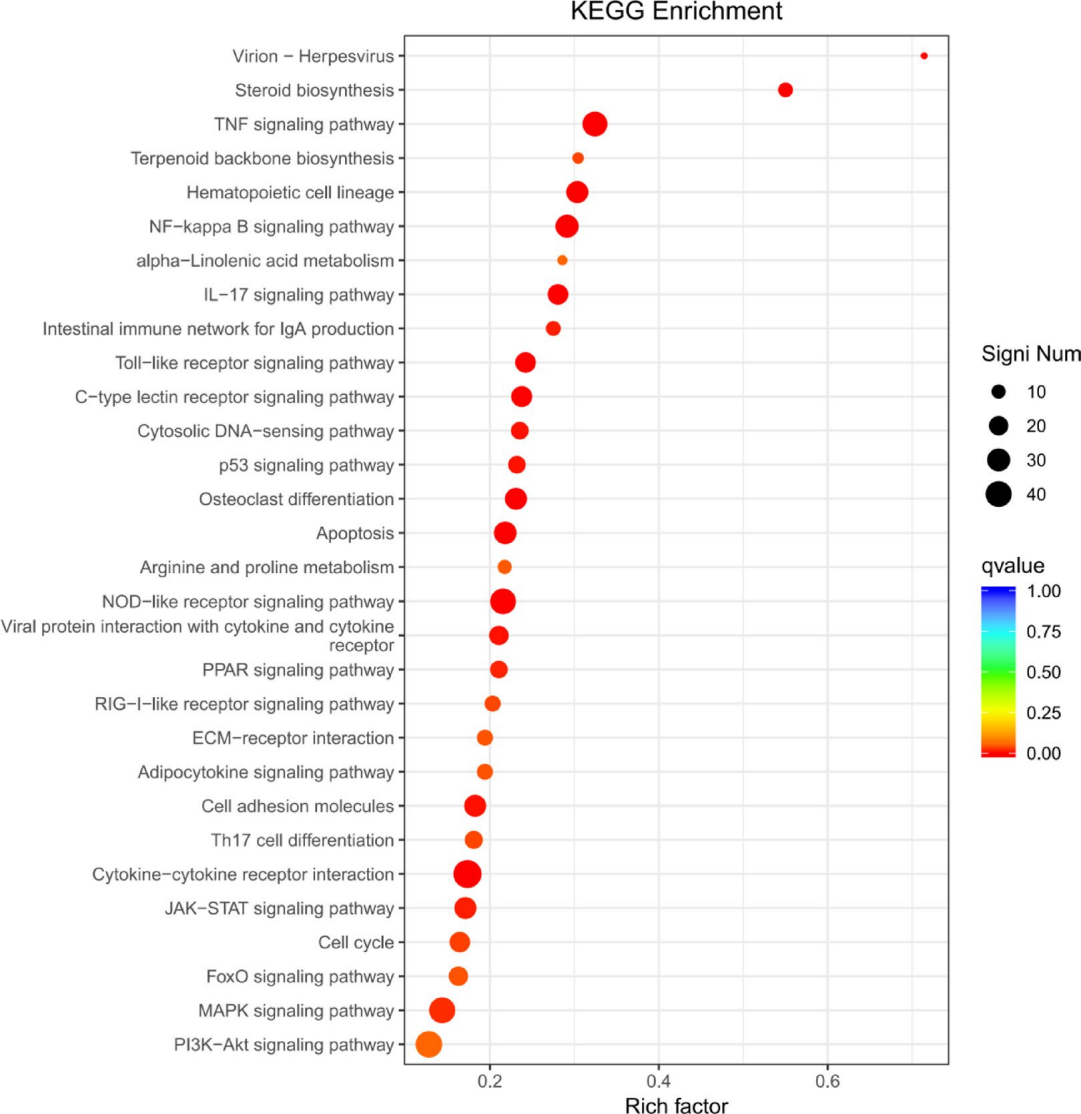
KEGG pathway enrichment results of differentially expressed genes from RAW 264.7 cells. KEGG imagery adapted from Kanehisa Laboratories^44^.

KEGG analysis showed that TNF (ko04668) and NF-κB (ko04064) were the most significantly immune-related enriched pathways in QsCATH-treated macrophages (*q*-value = 2.32 × 10^–10^ and 1.78 × 10^–7^, respectively), with coordinated downregulation of inflammatory mediators (*tnf*, *il6*, *ccl5*, *il1b*), which suggested a feedback-controlled immunity. Concurrently, NRL signaling (ko04621) modulated pathogen recognition (*nod1*/*2*, *nlrp3*), while IL-17 signaling (ko04657) suppression (*cxcl2*/*3*, *mmp9*) restrained neutrophil recruitment. Additional enrichment in cytokine-cytokine receptor interactions (*fas*) and apoptosis pathways (*bcl2l1*) highlighted broad immunomodulatory effects, balancing antimicrobial activity with inflammation resolution (Table 4; Fig. 2).

**Table 4:** KEGG pathway enrichment analysis of differentially expressed genes in RAW264.7 cells.

Pathway (KEGG ID)	*q*-value	DEGs with pathway annotation	Key DEGs (log_2_FoldChange)
TNF signaling (ko04668)	2.32 × 10^−10^	36	*tnf* (− 3.16), *il6* (− 12.40), *ccl5* (− 3.51), *cxcl2* (− 7.93)
NF-κB signaling (ko04064)	1.78 × 10^−7^	30	*nfkb1* (− 1.04), *tnf* (− 3.16), *il1b* (− 6.57)
NOD-like receptor (ko04621)	4.04 × 10^−6^	39	*nod1* (− 2.54), *nod2* (− 1.42), *nlrp3* (− 1.57)
IL-17 signaling (ko04657)	1.05 × 10^−5^	23	*cxcl2* (− 7.93), *cxcl3* (− 6.26), *mmp9* (− 1.76)
Cytokine-cytokine receptor interaction (ko04060)	6.70 × 10^−5^	48	*fas* (− 2.34)
Apoptosis (ko04210)	6.80 × 10^−5^	29	*bcl2l1* (− 2.12)

### GO analysis identifies QsCATH-mediated alterations in immune-related genes

**Fig. 3:**
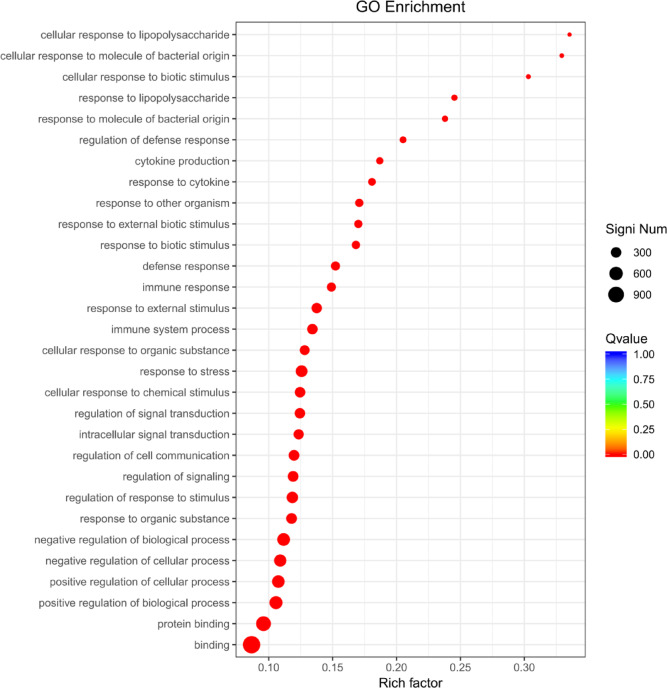
Gene ontology enrichment results of differentially expressed genes from RAW264.7 cells.

Transcriptomic analysis of macrophages treated with QsCATH revealed significant enrichment of immune-related GO terms. The most prominent terms were immune system process (GO:0,002,376), defense response (GO:0,006,952), and response to stimulus regulation (GO:0,048,583). Key genes associated with pathogen recognition, such as *tlr9*, *clec4n*, and *cd36*, were downregulated, alongside pro-inflammatory cytokines IL1B and IL6. Meanwhile, antimicrobial effectors, including LYZ1 and LYZ2, were upregulated, while nitric oxide synthase 2 (NOS2) showed reduced expression. Negative regulators of inflammation, such as SOCS3 and TNFAIP3, were suppressed, paralleled by the downregulation of NF-κB pathway components (RELB and NFKBIA). Terms related to stress responses, including response to oxidative stress (GO:0,006,950) and external stimulus (GO:0,009,605), highlighted modulation of GSTm5 and HSPA1A/B, suggesting altered redox homeostasis and endoplasmic reticulum stress adaptation (Table 5; Fig. 3).

**Table 5:** Immune-related GO terms in macrophages treated with QsCATH.

Gene ontology term	Q-value	DEGs with GO annotation	Key DEGs (log_2_FoldChange)
Immune system process (GO:0,002,376)	8.01 × 10⁻^25^	302	*tlr9* (-1.45), *cd40* (-3.18), *itgal* (-1.61), *cd86* (-1.48), *fas* (-2.34), *ccl3* (-3.04), *ccl4* (-2.14), *il1b* (-6.57), *il6* (-12.40)
Defense response (GO:0,006,952)	2.17 × 10⁻^22^	206	*nos2* (-6.13), *trpm2* (-2.65), *p2rx3* (-2.77), *ccl2* (-7.10), *cxcl3* (-6.26), *lyz1* (2.07), *lyz2* (1.49), *nlrp3* (-1.57), *casp4* (-2.24)
Regulation of response to stimulus (GO:0,048,583)	3.82 × 10⁻^22^	384	*socs3* (-2.06), *tnfaip3* (-1.97), *bcl2l1* (-2.12), *relb* (-1.23), *nfkbia* (-2.04), *nfkb1* (-1.04)
Response to stress (GO:0,006,950)	1.53 × 10⁻^2^⁶	413	*gstm5* (-11.21), *nqo1* (-1.37), *ern1* (-1.04), *hspa1b* (-2.30), *hspa1a* (-2.34)
Response to external stimulus (GO:0,009,605)	1.32 × 10⁻^25^	293	*clec4n* (-3.55), *clec4e* (-1.45), *tlr9* (-1.45), *cd36* (-1.18)
Negative regulation of biological process (GO:0,048,519)	1.32 × 10⁻^25^	515	*socs3* (-2.06), *tnfaip3* (-1.97), *bcl2l1* (-2.12), *nfkbia* (-2.04)

### Identification of putative QsCATH-interacting proteins and associated inflammatory signaling pathways

**Fig. 4:**
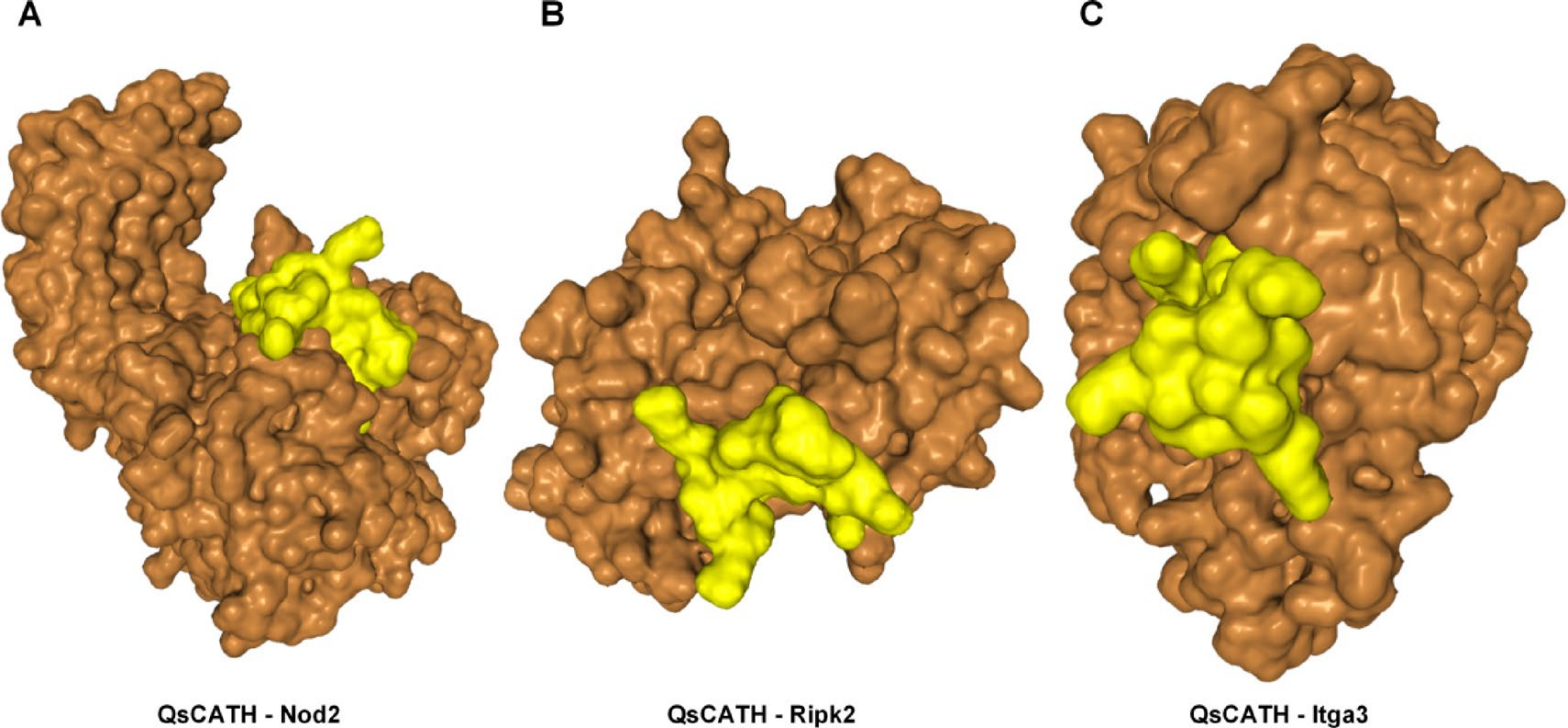
Molecular docking of QsCATH with Nod2, Ripk2, and Itga3 was conducted using HDOCK. Sequence sources/IDs: Itga3 (mouse) UniProt Q62470, Nod2 (mouse) UniProt Q8K3Z0, and Ripk2 (mouse) UniProt P58801. Models: SWISS-MODEL models (Nod2: Q8K3Z0_5irm.1.B; Ripk2: P58801_8 × 2o.1.B; Itga3: Q62470_8xen.1.A). Docking: HDOCK server. Full contact tables are provided in Table S1, 2, 3. The interaction between QsCATH and Nod2, Ripk2, and Itga3 is represented in the model using surface versions. Yellow represents QsCATH, and brown signifies three receptors.

Through differential gene expression analysis and pathway enrichment, three membrane-associated proteins were identified that potentially interact with QsCATH: Nod2, a bacterial peptidoglycan sensor linked to the NOD-like receptor pathway; Ripk2, a kinase mediating NF-κB activation downstream of NOD signaling; and Itga3, an integrin subunit implicated in cell adhesion. The interaction between QsCATH and Nod2, Ripk2, and Itga3 was examined using docking simulations on the HDOCK server, and the following models with binding and confidence scores were obtained: Nod2: -239.00, 0.86 (Fig. 4A); Ripk2: -213.24, 0.78 (Fig. 4B); Itga3: -290.08, 0.94 (Fig. 4C).

**Fig. 5:**
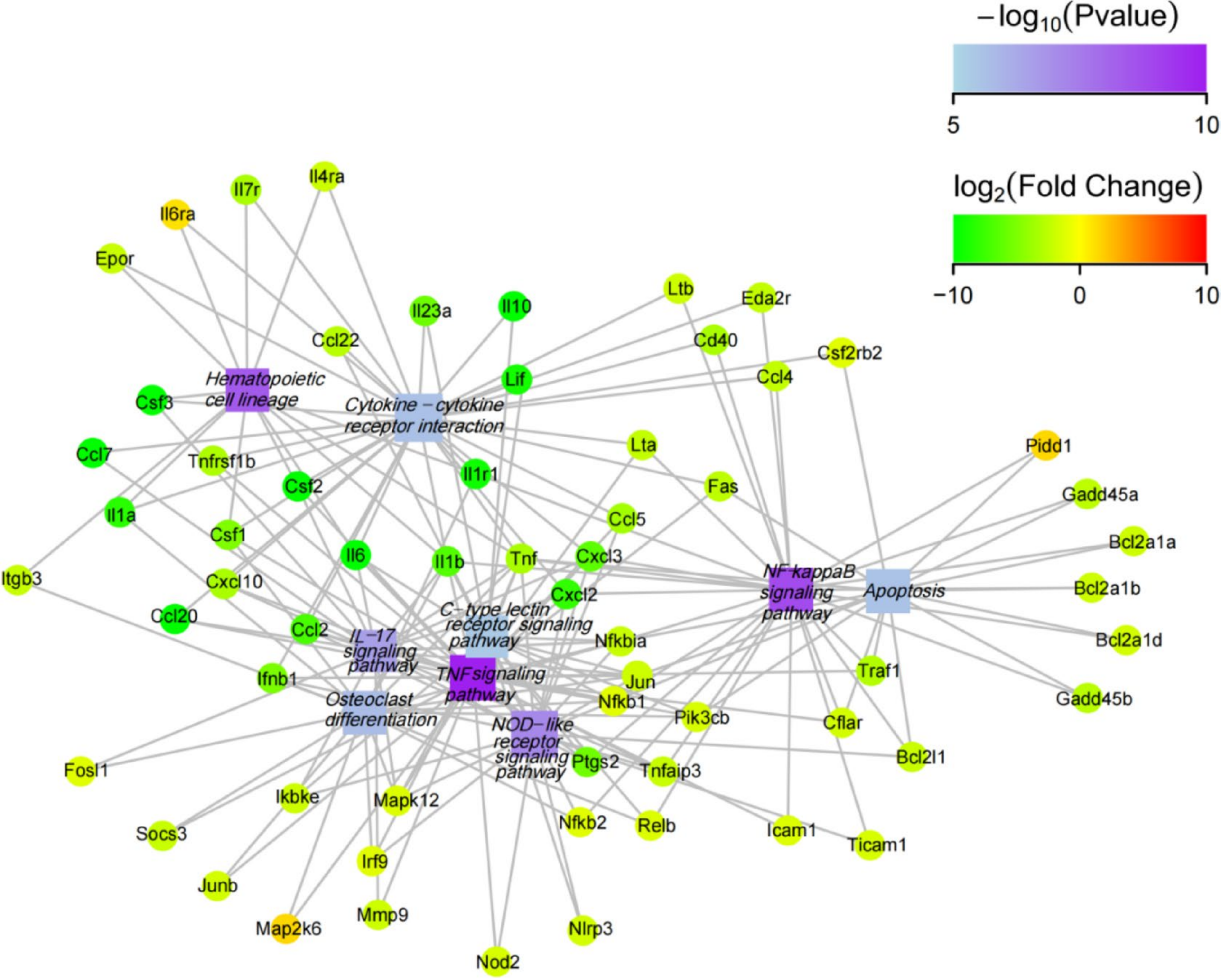
Top enriched KEGG pathways and associated gene interaction network. Square nodes represent functional information, circular nodes indicate genes, and lines show the associations between genes and functions. The square node color corresponds to the *p* value, with increasing color intensity reflecting an increasing degree of enrichment. The color intensity of circular nodes reflects log_2_(FoldChange) values (red: upregulation; green: downregulation).

Enriched pathways included the NOD-like receptor signaling pathway (ko04621), NF-κB signaling (ko04064), and TNF signaling (ko04668), with key genes (*tnf*, *il6*, *ccl5*) showing significant downregulation (|log2FC|> 3.0) (Fig. 5). Immune-related GO terms such as “immune system process” and “defense response” further highlighted suppression of inflammatory mediators (*il1b*, *cxcl2*, *nos2*) post-treatment.

### qRT-PCR verification of the RNA-seq results

**Fig. 6:**
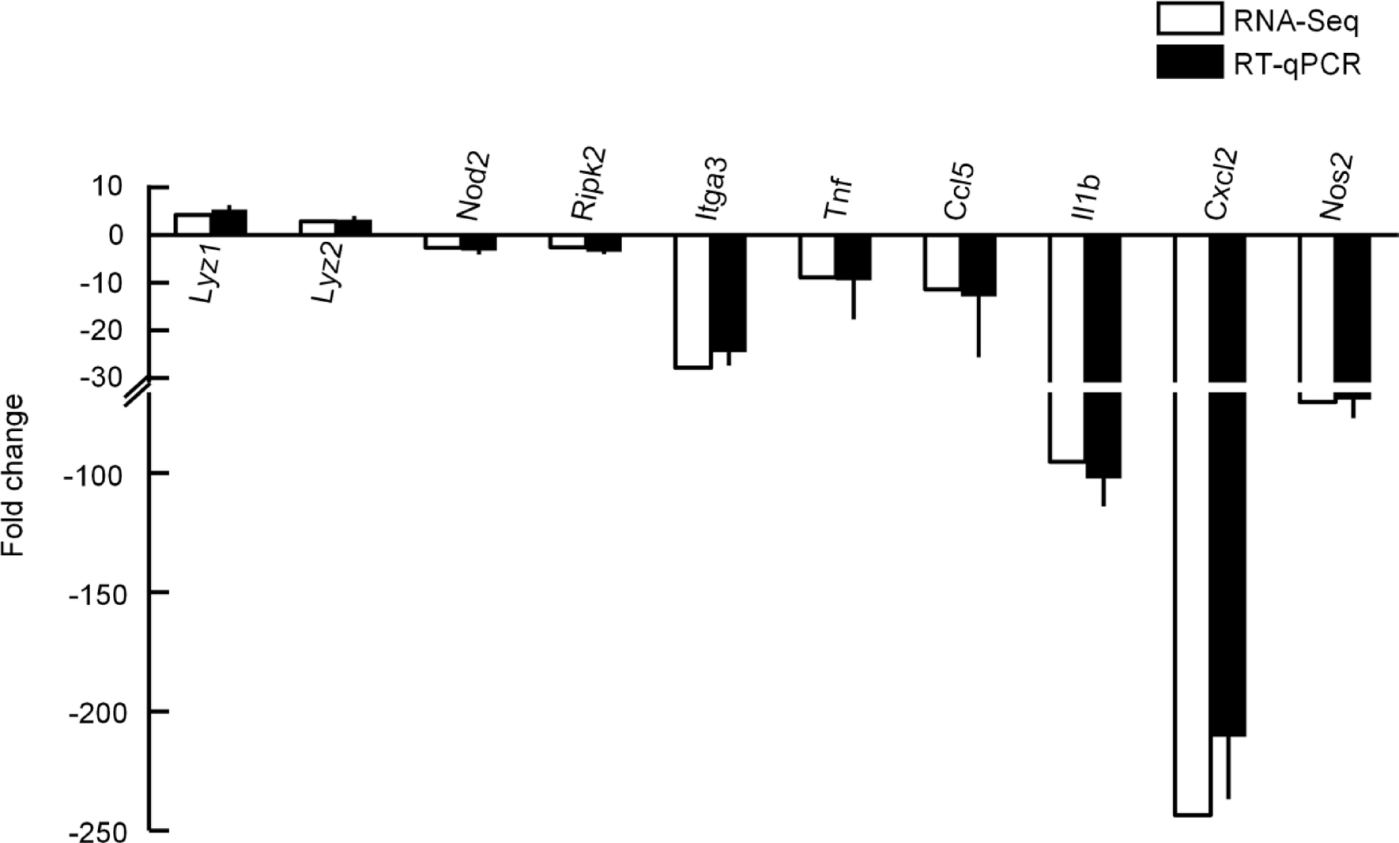
Validation of differentially expressed genes using quantitative polymerase chain reaction. Data are presented as the mean ± standard error of the mean for three samples.

To ascertain the reliability of the transcriptome sequencing results, quantitative polymerase chain reaction (qPCR) was conducted on a set of 10 DEGs, namely, *lyz1*, *nod2*, *ripk2*, *itga3*, *tnf*, *il6*, *ccl5*, *il1b*, *cxcl2*, and *nos2* (Fig. 6). The qPCR results revealed that the differential fold changes in the aforementioned 10 genes were consistent with the results obtained from transcriptome sequencing analysis. These validation results strongly show the reliability of the transcriptome sequencing analysis conducted in this study.

## Discussion

We observed a marked reduction of pro-inflammatory transcripts in RAW264.7 cells at 12 h after QsCATH treatment (e.g., *Tnf*, *Il6*, *Il1b*), accompanied upregulated bactericidal effectors (e.g., lysozymes LYZ1/2). Our finding that QsCATH reduces *Tnf*/*Il6*/*Il1b* transcripts in RAW264.7 cells at 12 h contrasts with Zheng et al., 2024^9^, who observed no change by qPCR. Given the documented sensitivity of RAW264.7 cytokine readouts to cell state/passaging^20^, trace endotoxin in culture reagents^21,22^, medium/vehicle composition^23^, and reference-gene normalization^24^, we interpret this discrepancy as reflecting context-dependent macrophage responses rather than a categorical conflict in biology.

QsCATH appears to show a “suppress-and-defend” profile. In our transcriptomic data, pro-inflammatory mediators dropped rapidly within 12 h of treatment (*Tnf* log_2_FC =  − 3.16; *Il6* =  − 12.40; *Il1b* =  − 6.57), while bactericidal effectors including lysozymes (*LYZ1*/*2*) were sustained (log_2_FC > 1.4). Notably, *Il10* decreased alongside *Tnf*/*Il1b* at 12 h. Because macrophage IL-10 depends on TLR–NF-κB/MAPK inputs and typically emerges as negative feedback to initial activation, upstream dampening by cathelicidins (e.g., LPS neutralization) predicts a parallel reduction of both pro- and counter-regulatory cytokines under these conditions^25,26^. This pattern aligns with prior cathelicidin studies showing dampening of excessive outputs without enforcing a uniform M2 switch: human LL-37 reduces TNF-α in both M1- and M2-polarized macrophages while preserving key functions such as phagocytosis and efferocytosis, i.e., tempering rather than repolarizing^27^. CATH-2/LL-37 preferentially inhibit M1 activation (with milder effects on M2) and CATH-2 also suppresses activation by whole bacteria, underscoring selective, stimulus-dependent modulation^28^. In specific contexts, cathelicidins can tilt toward M2-like features—for example cathelicidin-WA in RAW264.7 cells challenged with *E. coli* K88^29^. Amphibian cathelicidins similarly suppress NF-κB/MAPK-driven cytokines (e.g., tree-frog cathelicidin-PP)^30^ and can orchestrate phase-specific resolution during wound repair (e.g., BugaCATH in mice)^31^. Given that amphibian skin is a permeable, front-line immune organ continuously exposed to microbes, a fast “control-inflammation-while-defending” strategy is plausibly adaptive in ectotherms^32,33^. Accordingly, we will quantify canonical M1/M2 markers, perform flow-cytometric phenotyping, and profile cytokines to determine whether QsCATH biases polarization or predominantly tempers activation (rather than enforcing a classical M2 switch).

Molecular docking predictions revealed that QsCATH may interact with multiple immune signaling components, such as the membrane-bound integrin Itga3 (binding score: -290.08) and intracellular proteins Nod2 (-239.00) and Ripk2 (-213.24). These computational results indicate potential binding affinities; Across receptors, the top HDOCK poses place the cationic, amphipathic α-helical face of QsCATH on acidic/polar receptor surfaces, consistent with cathelicidin biophysics^34,35^. Site mapping indicates proximity to laminin-engaging headpiece surfaces on α3β1 integrin, the concave LRR MDP site on NOD2^36,37^, and a non-ATP lateral surface on RIPK2—supporting interface/allosteric modulation rather than ATP-site competition^36–40^. Transcriptomic data corroborate the functional relevance of these predictions, showing coordinated downregulation of Nod1/2 (− 2.54/− 1.42 log_2_FC), Ripk2 (− 1.98), and NF-κB pathway genes (Nfkb1: − 1.04, Relb: − 1.23). This multi-target suppression pattern reveals a synergistic mechanism wherein QsCATH disrupts ligand sensing (via Itga3-mediated endocytosis modulation) and intracellular signal propagation (via NOD-NF-κB axis inhibition). However, the exact molecular interplay between these targets requires further investigation.

A striking feature of the immunomodulatory activity of QsCATH is its concurrent suppression of pro-inflammatory cytokines (*Tnf*, *Il1b*) and negative feedback regulators (*SOCS3*, *TNFAIP3*). This dual inhibition could represent a novel “threshold control” mechanism, wherein acute inflammation is rapidly curtailed while baseline antimicrobial defenses remain active, a critical adaptation for organisms lacking adaptive immune redundancy. The transcriptional repression of oxidative stress genes (*GSTm5*: − 11.21 log_2_FC, *NQO1*: − 1.37) further implies metabolic reprogramming, potentially reallocating resources toward microbial clearance. These findings align with those of recent studies on amphibian AMPs, which emphasize their role as multifunctional sentinels at the host-environment interface^10,41^.

The findings from this study provide compelling evidence for the immunomodulatory potential of QsCATH; nevertheless, some limitations should be acknowledged. First, the in vitro macrophage model lacks the complexity of tissue-resident immune populations and pathogen-challenged microenvironments. Second, predicted receptor interactions, though supported by transcriptomic trends, require biochemical validation. We will determine kinetics and affinity of QsCATH binding to Itga3 ectodomain and Nod2-LRR by SPR/BLI (with ITC to define thermodynamics); (2) perform co-immunoprecipitation to test whether QsCATH disrupts the Nod2–Ripk2 complex; and (3) evaluate functional necessity via CRISPR-Cas9 knockout of Itga3, Nod2, or Ripk2, quantifying NF-κB–luciferase activity and Tnf/Il6/Il1b expression. These assays will determine whether QsCATH directly engages these targets and whether such engagement is required for suppression of NOD–NF-κB signaling.

## Conclusion

The study results elucidate the multitarget immunomodulatory mechanism of the QsCATH in macrophages. Transcriptomics reveals that QsCATH suppresses pro-inflammatory genes (*Tnf, Il6, Il1b*; log_2_FC < − 3.0) and the NF-κB/NOD pathways and enhances antimicrobial effectors (LYZ1/2), thereby achieving a “balanced inhibition” of inflammation without compromising host defense. Molecular docking was used to predict interactions with membrane integrin Itga3 and intracellular nodes Nod2/Ripk2, suggesting dual targeting of ligand endocytosis and signal transduction. This multitarget strategy challenges the mammalian TLR4-centric paradigm, offering novel insights into amphibian immune evolution. Future work should validate receptor interactions through gene-editing models and explore the therapeutic potential of QsCATH as a dual anti-inflammatory/anti-infective agent.

## Methods

### Cell culture and experimental procedures

RAW264.7 murine macrophages were cultured in Dulbecco’s Modified Eagle Medium (Gibco, USA) containing 10% heat-inactivated fetal bovine serum (HyClone, USA) and antibiotic–antimycotic solution (1% v/v, Sigma-Aldrich). Cells were maintained at 37 °C in a humidified 5% CO_2_ incubator. For experimental treatments, cells were treated with QsCATH (mature cathelicidin peptide) at 1.0 μg/mL for 12 h. The peptide was produced by Fmoc-based solid-phase synthesis, purified by RP-HPLC (≥ 95% purity), and its identity verified by mass spectrometry^9^. Post-treatment, total RNA was isolated from both QsCATH-treated and control groups using TRIzol reagent (Invitrogen, USA) following the manufacturer’s protocol. Each experimental condition included three independent biological replicates. RNA concentration and purity were determined through fluorescence spectroscopy using a Qubit 4.0 system (Thermo Fisher Scientific, Waltham, MA, USA), with triplicate measurements performed for each sample.

### Library construction and transcriptome analysis

Sequencing libraries were prepared using the SureSelect XT HS2 mRNA Library Preparation Kit (Agilent Technologies, USA), followed by fragment size selection with AMPure XP magnetic beads (Beckman Coulter, USA). High-throughput paired-end sequencing (2 × 150 bp) was performed on an MGISEQ-2000RS platform (MGI) using sequencing-by-synthesis chemistry. Raw sequencing data (BCL format outputs) were converted to demultiplexed FASTQ files using bcl2fastq v2.20 (Illumina, USA). Sequence preprocessing included (1) adapter removal and quality trimming via Trimmomatic v0.36 (sliding window) and (2) read quality validation using FASTQC v0.11.9. Clean reads were aligned to the *Mus musculus* reference genome using HISAT2 v2.2.1 with default parameters.

Transcript quantification and differential expression analysis were conducted using a multi-step workflow: StringTie v2.1.4 was employed to assemble transcripts and estimate abundance, followed by statistical analysis using DESeq2 v1.28.1 with genomic bias correction. Differentially expressed genes (DEGs) were functionally enriched using clusterProfiler v3.6. To enhance analytical rigor, transcripts meeting either of the following criteria were excluded: (1) expression levels < 0.3 TPM in treatment and control groups, or (2) insufficient read coverage (< 10 reads per transcript). Significant DEGs were identified based on a Benjamini–Hochberg adjusted *p*-value < 0.05 and absolute log_2_-transformed fold change > 1 (equivalent to > 2.0-fold difference in linear scale).

### Functional enrichment analysis

Gene Ontology (GO) annotation and Kyoto Encyclopedia of Genes and Genomes (KEGG) pathway analyses were systematically conducted to interpret the biological relevance of DEGs. The complete set of annotated genes from the *M. musculus* genome was the reference background. Functional categorization was performed through three GO domains: biological processes, molecular functions, and cellular components, using the clusterProfiler v3.6 package (R/Bioconductor) with a hypergeometric test. Pathway enrichment analysis was performed using KEGG (https://www.genome.jp/kegg/), a publicly available pathway-related database.

### Computational protein interaction prediction

Molecular docking simulations were performed using the HDOCK server (http://hdock.phys.hust.edu.cn/) to investigate potential binding interactions between QsCATH and key signaling proteins (Nod2, Ripk2, and Itga3)^42^. The default parameters were used for docking. Structural models were generated using canonical amino acid sequences retrieved from the National Center for Biotechnology Information (NCBI) GenBank database: QsCATH (WOZ29869), Nod2 (AAN84594), Ripk2 (AAH69878), and Itga3 (AAH62205).

### *Transcriptomic validation *via* reverse transcription quantitative PCR*

To validate RNA sequencing results, independent verification was performed using SYBR Green-based quantitative polymerase chain reaction (PCR). Total RNA was isolated from cellular lysates using TRIzol reagent (Invitrogen, USA), followed by cDNA synthesis with the PrimeScript RT Master Mix (Perfect Real Time, TaKaRa Bio, Dalian, China) containing AMV reverse transcriptase. Amplification reactions were conducted in technical quadruplicates using a CFX96 Touch Real-Time PCR System (Bio-Rad Laboratories, Hercules, CA, USA) under standardized cycling conditions: initial denaturation at 95 °C for 30 s, annealing/extension using 40 cycles of 95 °C for 5 s, and 60 °C for 30 s, with melt curve analysis at 65–95 °C, with 0.5 °C increments. Primer specificity was confirmed through agarose gel electrophoresis and melt curve analysis (single peak verification). 18S rRNA was used as the endogenous control for normalization. The primers used in the experiments are listed in Table 1. The 2^−ΔΔCt^ method was used for relative quantification^43^, with three biological replicates per condition and four technical replicates per sample.

## Supplementary Information

Supplementary Information.

## Data Availability

The transcriptome data was submitted to NCBI Sequence Read Archive (SRA) database under accession number PRJNA1220286. The datasets used and/or analysed during the current study are available from the corresponding author on reasonable request.
