# Supplementary material for: Immunomodulatory effects of QsCATH on macrophages: transcriptomic insights and molecular docking analysis
Source: Sci Rep. 2025 Dec 12;15:43728. doi: 10.1038/s41598-025-28482-9 (PMC12700900; doi:10.1038/s41598-025-28482-9)
Supplement: Supplementary file 1 — Supplementary Information. [file 41598_2025_28482_MOESM1_ESM.docx]

**Supplementary Table 1.**

MmNod2-QsCATH interface residue pairs

| MmNod2  interface residues | QsCATH  interface residues | MmNod2-QsCATH interface residue pairs |
| --- | --- | --- |
| ALA 241A | CYS 7A | 241A - 8A |
| GLN 243A | ARG 8A | 243A - 8A |
| PHE 359A | GLY 9A | 359A - 14A |
| PHE 361A | ILE 10A | 361A - 16A |
| ARG 364A | PHE 11A | 361A - 17A |
| PRO 400A | CYS 12A | 364A - 19A |
| ASP 401A | ARG 13A | 400A - 10A |
| ALA 402A | ARG 14A | 401A - 10A |
| VAL 403A | GLY 16A | 401A - 11A |
| SER 404A | SER 17A | 401A - 13A |
| ALA 405A | SER 19A | 402A - 10A |
| LEU 406A | LEU 20A | 402A - 12A |
| ARG 408A | GLY 22A | 403A - 10A |
| GLN 637A | ARG 23A | 403A - 11A |
| LYS 638A | ALA 25A | 403A - 12A |
| ALA 639A | LYS 26A | 404A - 10A |
| GLU 640A | ASP 27A | 404A - 11A |
| PRO 641A |  | 404A - 12A |
| ILE 695A |  | 404A - 13A |
| PRO 696A |  | 405A - 10A |
| ALA 698A |  | 405A - 11A |
| VAL 699A |  | 405A - 12A |
| PRO 700A |  | 405A - 20A |
| THR 703A |  | 406A - 19A |
| LYS 704A |  | 406A - 20A |
| SER 705A |  | 408A - 10A |
| MET 706A |  | 637A - 14A |
| HIS 707A |  | 638A - 14A |
| PRO 710A |  | 639A - 14A |
|  |  | 640A - 14A |
|  |  | 641A - 13A |
|  |  | 641A - 14A |
|  |  | 695A - 26A |
|  |  | 695A - 27A |
|  |  | 696A - 26A |
|  |  | 698A - 11A |
|  |  | 698A - 13A |
|  |  | 699A - 13A |
|  |  | 699A - 14A |
| MmNod2  interface residues | QsCATH  interface residues | MmNod2-QsCATH interface residue pairs |
|  |  | 700A - 13A |
|  |  | 700A - 14A |
|  |  | 703A - 13A |
|  |  | 704A - 13A |
|  |  | 705A - 13A |
|  |  | 706A - 11A |
|  |  | 706A - 22A |
|  |  | 706A - 23A |
|  |  | 706A - 25A |
|  |  | 706A - 26A |
|  |  | 706A - 27A |
|  |  | 707A - 7A |
|  |  | 707A - 8A |
|  |  | 707A - 9A |
|  |  | 707A - 27A |
|  |  | 710A - 13A |

**Supplementary Table 2.**

MmRipk2-QsCATH interface residue pairs

| MmRipk2  interface residues | QsCATH  interface residues | MmRipk2-QsCATH interface residue pairs |
| --- | --- | --- |
| ARG 74A | ARG 3A | 74A - 8A |
| PHE 75A | LYS 4A | 75A - 8A |
| SER 76A | PRO 5A | 76A - 6A |
| TYR 77A | PRO 6A | 76A - 8A |
| PRO 119A | CYS 7A | 76A - 9A |
| LEU 120A | ARG 8A | 77A - 8A |
| PHE 122A | GLY 9A | 77A - 9A |
| ARG 123A | ILE 10A | 77A - 10A |
| HIS 126A | PHE 11A | 119A - 12A |
| GLU 127A | CYS 12A | 119A - 14A |
| ASP 155A | ARG 13A | 120A - 12A |
| ASN 156A | ARG 14A | 120A - 15A |
| GLU 157A | VAL 15A | 120A - 19A |
| PHE 158A | SER 19A | 120A - 20A |
| HIS 159A | LEU 20A | 122A - 11A |
| LYS 161A | ILE 21A | 122A - 12A |
| PHE 297A |  | 122A - 13A |
| GLU 298A |  | 122A - 14A |
| ASP 299A |  | 123A - 10A |
| ILE 300A |  | 123A - 11A |
| THR 301A |  | 123A - 12A |
| GLN 308A |  | 123A - 20A |
| ALA 312A |  | 123A - 21A |
| LYS 313A |  | 126A - 10A |
| ILE 314A |  | 127A - 10A |
| ALA 365A |  | 155A - 3A |
| PRO 366A |  | 155A - 4A |
|  |  | 156A - 3A |
|  |  | 157A - 3A |
|  |  | 157A - 4A |
|  |  | 157A - 5A |
|  |  | 157A - 6A |
|  |  | 157A - 20A |
|  |  | 157A - 21A |
|  |  | 158A - 19A |
|  |  | 158A - 20A |
|  |  | 158A - 21A |
|  |  | 159A - 5A |
|  |  | 159A - 6A |
| MmRipk2  interface residues | QsCATH  interface residues | MmRipk2-QsCATH interface residue pairs |
|  |  | 159A - 10A |
|  |  | 159A - 20A |
|  |  | 159A - 21A |
|  |  | 161A - 6A |
|  |  | 297A - 13A |
|  |  | 297A - 14A |
|  |  | 298A - 10A |
|  |  | 298A - 13A |
|  |  | 299A - 13A |
|  |  | 300A - 13A |
|  |  | 301A - 10A |
|  |  | 301A - 13A |
|  |  | 308A - 8A |
|  |  | 312A - 8A |
|  |  | 313A - 7A |
|  |  | 314A - 8A |
|  |  | 365A - 4A |
|  |  | 366A - 4A |

**Supplementary Table 3.**

MmItga3-QsCATH interface residue pairs

| MmItga3  interface residues | QsCATH  interface residues | MmItga3-QsCATH interface residue pairs |
| --- | --- | --- |
| HIS 111A | ARG 3A | 111A - 16A |
| ARG 112A | LYS 4A | 111A - 17A |
| MET 127A | PRO 5A | 111A - 18A |
| ASP 159A | PRO 6A | 112A - 17A |
| MET 165A | ILE 10A | 127A - 18A |
| GLN 167A | PHE 11A | 127A - 21A |
| THR 170A | CYS 12A | 159A - 3A |
| PRO 184A | ARG 13A | 165A - 21A |
| GLY 185A | ARG 14A | 167A - 17A |
| ASN 188A | VAL 15A | 167A - 18A |
| TRP 189A | GLY 16A | 167A - 19A |
| ASN 216A | SER 17A | 170A - 17A |
| TYR 218A | GLY 18A | 170A - 19A |
| TYR 221A | SER 19A | 184A - 17A |
| ARG 244A | LEU 20A | 184A - 19A |
| HIS 247A | ILE 21A | 185A - 19A |
| GLY 274A |  | 188A - 3A |
| ALA 275A |  | 189A - 3A |
| TYR 276A |  | 189A - 21A |
| TYR 300A |  | 216A - 3A |
| PHE 302A |  | 218A - 19A |
| VAL 308A |  | 218A - 20A |
| ILE 341A |  | 218A - 21A |
| PHE 363A |  | 221A - 12A |
| LEU 397A |  | 221A - 15A |
| THR 399A |  | 221A - 19A |
| TYR 402A |  | 221A - 20A |
| SER 424A |  | 244A - 4A |
|  |  | 244A - 5A |
|  |  | 244A - 6A |
|  |  | 244A - 20A |
|  |  | 244A - 21A |
|  |  | 247A - 6A |
|  |  | 274A - 10A |
|  |  | 275A - 10A |
|  |  | 276A - 10A |
|  |  | 276A - 11A |
|  |  | 276A - 12A |
|  |  | 276A - 20A |
| MmItga3  interface residues | QsCATH  interface residues | MmItga3-QsCATH interface residue pairs |
|  |  | 300A - 10A |
|  |  | 300A - 11A |
|  |  | 300A - 12A |
|  |  | 300A - 13A |
|  |  | 300A - 14A |
|  |  | 302A - 10A |
|  |  | 308A - 10A |
|  |  | 341A - 12A |
|  |  | 363A - 13A |
|  |  | 363A - 14A |
|  |  | 397A - 14A |
|  |  | 399A - 14A |
|  |  | 402A - 14A |
|  |  | 402A - 15A |
|  |  | 402A - 16A |
|  |  | 424A - 14A |
